# Taxonomy of the genus *Peyerimhoffia* Kieffer from Mainland China, with a description of seven new species (Diptera, Sciaridae)

**DOI:** 10.3897/zookeys.382.4948

**Published:** 2014-02-20

**Authors:** Kai Shi, Junhao Huang, Sujiong Zhang, Hong Wu

**Affiliations:** 1 Institute of Forestry Protection, School of Forestry and Biotechnology, Zhejiang A & F University, 88 Huancheng Beilu, Linan, Hangzhou, Zhejiang 311300, China; 2 Forestry Bureau of Pan’an County, Pan’an 322300, Zhejiang, China

**Keywords:** Diptera, Sciaridae, new species, new record, China

## Abstract

The taxonomy of the genus *Peyerimhoffia* Kieffer in China was studied. Eight species were recognized, including seven new species that are herein described and illustrated: *P. hamata* **sp. n.**, *P. obesa* **sp. n.**, *P. sparsula* **sp. n.**, *P. longiprojecta* **sp. n.**, *P. brachypodua* **sp. n.**, *P. yunnana* **sp. n.**, and *P. shennongjiana* **sp. n.** In addition, *P. vagabunda* (Winnertz, 1867) is reported for the first time from China. A key to these Chinese species is provided.

## Introduction

*Peyerimhoffia* Kieffer, 1903 was described as a monotypic genus (type species *Peyerimhoffia brachyptera* Kieffer, 1903 = *Sciara vagabunda* Winnertz, 1867). Tuomikoski (1960) regarded the taxon as a subgenus within *Plastosciara* Berg, 1899 = *Cratyna* Winnertz, 1867 (type species *Cratyna atra* Winnertz), followed by Mohrig and Mamaev (1974) and Menzel and Mohrig (1998, 2000). However, current phylogeny study based on 64 morphological characteristics of adult males (Vilkamaa and Hippa 2005) suggests that *Peyerimhoffia* deserves a generic status.

We herein follow the redefined concept of *Peyerimhoffia* from Vilkamaa and Hippa (2005). The taxon is similar to *Mohrigia* and *Cratyna (Spathobdella)* Frey in having a group of setae inside the gonostylus, a visible aedeagal margin of the tegmen, and in having slightly elongated necks of antennal flagellomeres. *Peyerimhoffia* differs in having strongly elongated dorsomesial setae on the gonostylus, and in having strongly angulate margin of tegmen. The species earlier placed in *Peyerimhoffia* and the species of the *Corynoptera crassistylata* group sensu Menzel and Mohrig (2000) proved to form a monophyletic group in two cladistic analyses using adult morphological characters (Vilkamaa and Hippa 2004, Hippa and Vilkamaa 2005). In the latter, the monophyly was supported by five character states, two of which unique: “Mesial side of gonostylus with additional elongated setae” and “apicoventral part of gonostylus with nonsetose area” (Hippa and Vilkamaa 2005).

The concept of *Peyerimhoffia* sensu Vilkamaa & Hippa was crisized by Menzel et al. (2011) but without any argumentation or analysis. A recent molecular phylogeny of Shin et al. (2013) placed *Spathobdella* and *Peyerimhoffia* as sister groups, but of *Peyerimhoffia*, only the type species was in the ingroup of the analysis. Accordingly, there is no molecular evidence against the monophyly of *Peyerimhoffia* in the present sense.

The genus has never been recorded from China. In this study, we taxonomically revise the genus based on specimens collected in recent years by Zhejiang A&F University, China. Detailed illustrations, differential diagnoses, distributional information of each species, and a key to the Chinese species are provided.

## Material and methods

All specimens were collected by sweeping, malaise trapping, and yellow trapping and were preserved in 75% ethanol. All were mounted on microscope slides in xylol-based Canada balsam after clearing in xylol. The slides were made under a Nikon SMZ1500 stereo microscope. The specimens were observed, measured, and illustrated under a Leica DM2500 microscope. This study was based on males only because most species characteristics of *Peyerimhoffia* are based on the male morphology, whereas females are not generally identifiable to the species level. The terminology follows Vilkamaa and Hippa (2005). All of the type specimens in this study were deposited at the Institute of Forest Protection, Zhejiang A&F University, Hangzhou, Zhejiang Province, China (ZAFU).

## Taxonomy

### Key to the *Peyerimhoffia* species from China (males)

**Table:** 

1	Maxillary palp 1-segmented (Figs 1C, 2C)	2
–	Maxillary palp 3-segmented (Figs 3C, 4C, 5C, 6C, 7C)	4
2	Hypopygium with a lobe-like projecting intercoxal area, tegmen slightly and smoothly curved and sclerotized (Fig. 1B)	*Peyerimhoffia hamata* sp. n.
–	Hypopygium without lobe-like projecting intercoxal area, tegmen strongly curved and sclerotized (Fig. 2B)	3
3	Gonostylus very tumid, broadest on apical part, apical tooth short (about half as long as width of gonostylus) (Fig. 2A)	*Peyerimhoffia obesa* sp. n.
–	Gonostylus slightly tumid, broadest on mesial part, apical tooth long (as long as width of gonostylus)	*Peyerimhoffia vagabunda* (Winnertz, 1867)
4	Gonostylus narrowed, without apical lobe except a tooth on its apex (Figs 3A, 4B)	5
–	Gonostylus inflated, with an distinct apical lobe bearing a tooth (Figs 5A, 6A, 7A)	6
5	Apical tooth long (as long as width of gonostylus), tegmen almost truncate on the apical margin with a weak process (Fig. 3A, B)	*Peyerimhoffia sparsula* sp. n.
–	Apical tooth short (about half as long as width of gonostylus), tegmen greatly projected in the middle of apical margin (Fig. 4A, B)	*Peyerimhoffia longiprojecta* sp. n.
6	Basal palpomere with a sensory pit and one seta, gonostylus relatively narrowed, with apex densely setose (Fig. 5A, C)	*Peyerimhoffia brachypoda* sp. n.
–	Basal palpomere without sensory pit and with four setae, gonostylus inflated, with apex sparsely setose or bare (Figs 6A, C, 7A, C)	7
7	Apex of the gonostylus sparsely setose, and its apical tooth light and not sclerotized, tegmen narrowed abruptly on the middle and almost equilateral in ventral view (Fig. 6A, B)	*Peyerimhoffia yunnana* sp. n.
–	Apex of the gonostylus bare, and its apical tooth dark and sclerotized, tegmen narrowed evenly and almost triangular in ventral view (Fig. 7A, B)	*Peyerimhoffia shennongjiana* sp. n.

#### Peyerimhoffia hamata

Shi & Huang sp. n.

http://zoobank.org/84B856A9-D5D6-45CB-9125-2118E425E230

http://species-id.net/wiki/Peyerimhoffia_hamata

Figs 1 , 8A , 9

##### Specimens examined.

*Holotype*, male. CHINA. Zhejiang Province, Linan, Jincheng, Mt. Xijingshan, sweep-net, 29.IV.2011, Kai Shi [SM01563]. *Paratype*, ZHEJIANG. 1 male, Qingyuan, Mt. Baishanzu, Wanli-linchang, sweep-net, 24.VII.2012, Lu-Jing Yang [SM01732].

##### Description (male).

**Figure 1.**
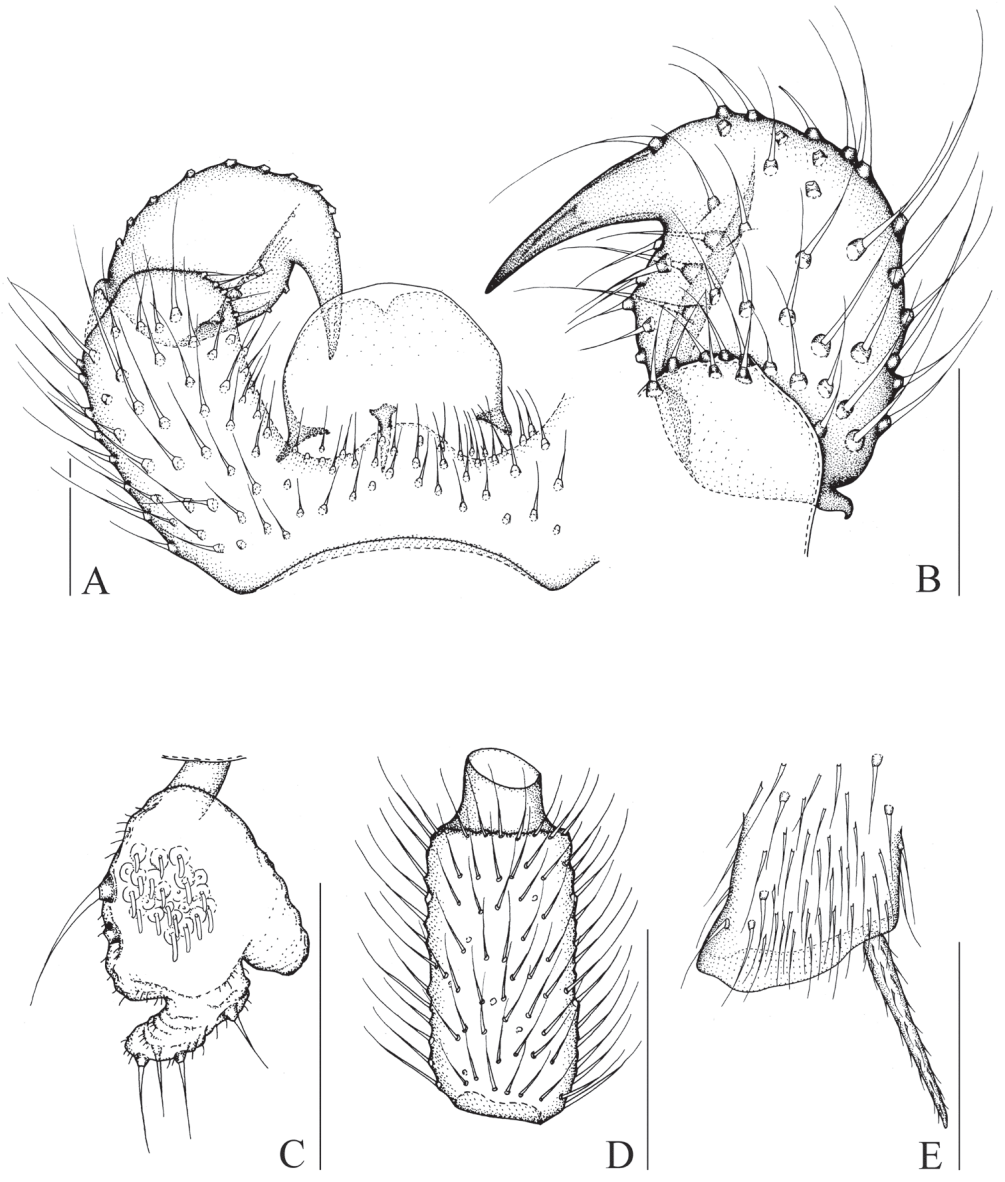
*Peyerimhoffia hamata* Shi & Huang, sp. n., male, holotype. **A** Part of hypopygium, ventral view **B** Right gonostylus, ventral view **C** Palp, lateral view **D** Fourth flagellomere, lateral view **E** Apex of foretibia, prolateral view. Scale, 0.10 mm.

Head dark brown; antenna, thorax, abdomen, and hypopygium brown; palp pale brown; legs pale yellowish-brown; wings fumose. **Head** (Fig. 1C, D). Eye bridge with 3 rows of facets. Prefrons with 4 setae. Clypeus non-setose. Maxillary palp 1-segmented, with 6 setae. Length/width of fourth flagellomere: 2.07–2.16. **Thorax.** Anterior pronotum with 3 setae, episternum 1 with 3 setae. **Wings** (Fig. 8A). Wing length 1.88–2.21 mm, width/length: 0.38–0.41. c/w: 0.61–0.73. R1/R: 0.68–0.90. M, Cu, stM, and r-m non-setose. **Legs** (Fig. 1E). Front tibia with non-bordered prolateral patch of modified setae. Length of spur/width of foretibia 1.18–1.23. Length of femur/length of metatarsus: foreleg 1.24–1.38. Length of metatarsus/length of tibia: foreleg 0.49–0.56, hind leg 0.44–0.49. Length of hind tibia/length of thorax 1.30–1.42. **Hypopygium** (Fig. 1A, B). Sternite 10 with one seta on each half.

##### Distribution.

China, Zhejiang (Fig. 9).

##### Remarks.

Based on the form of the gonostylus, the new species is similar to *Peyerimhoffia vagabunda* (Winnertz, 1867). However, the new species can be distinguished in having an irregularly shaped palp (Fig. 1C), the gonostylus distinctly and mesially constricted on dorsal side, the tegmen slightly and smoothly curved, and a lobe-like projecting intercoxal area on the hypopygium. In contrast, *Peyerimhoffia vagabunda* has a regularly shaped palp, the gonostylus is evenly rounded on the dorsal side, the tegmen is strongly curved, and no lobe-like projecting intercoxal area occurs on the hypopygium.

##### Etymology.

This species is named after its hook-like gonostylus (Latin adjective *hamatus* = hooked).

#### Peyerimhoffia obesa

Shi & Huang sp. n.

http://zoobank.org/FD68BA82-6A0C-4B21-8516-C7B9DFE8BA27

http://species-id.net/wiki/Peyerimhoffia_obesa

Figs 2 , 8B , 9

##### Specimens examined.

*Holotype*, male. CHINA. Shanxi Province, Qinshui, Xiachuancun, Fuyuhe, sweep-net, 26.VII.2012, Kai Shi [SM01795].

##### Description (male).

**Figure 2.**
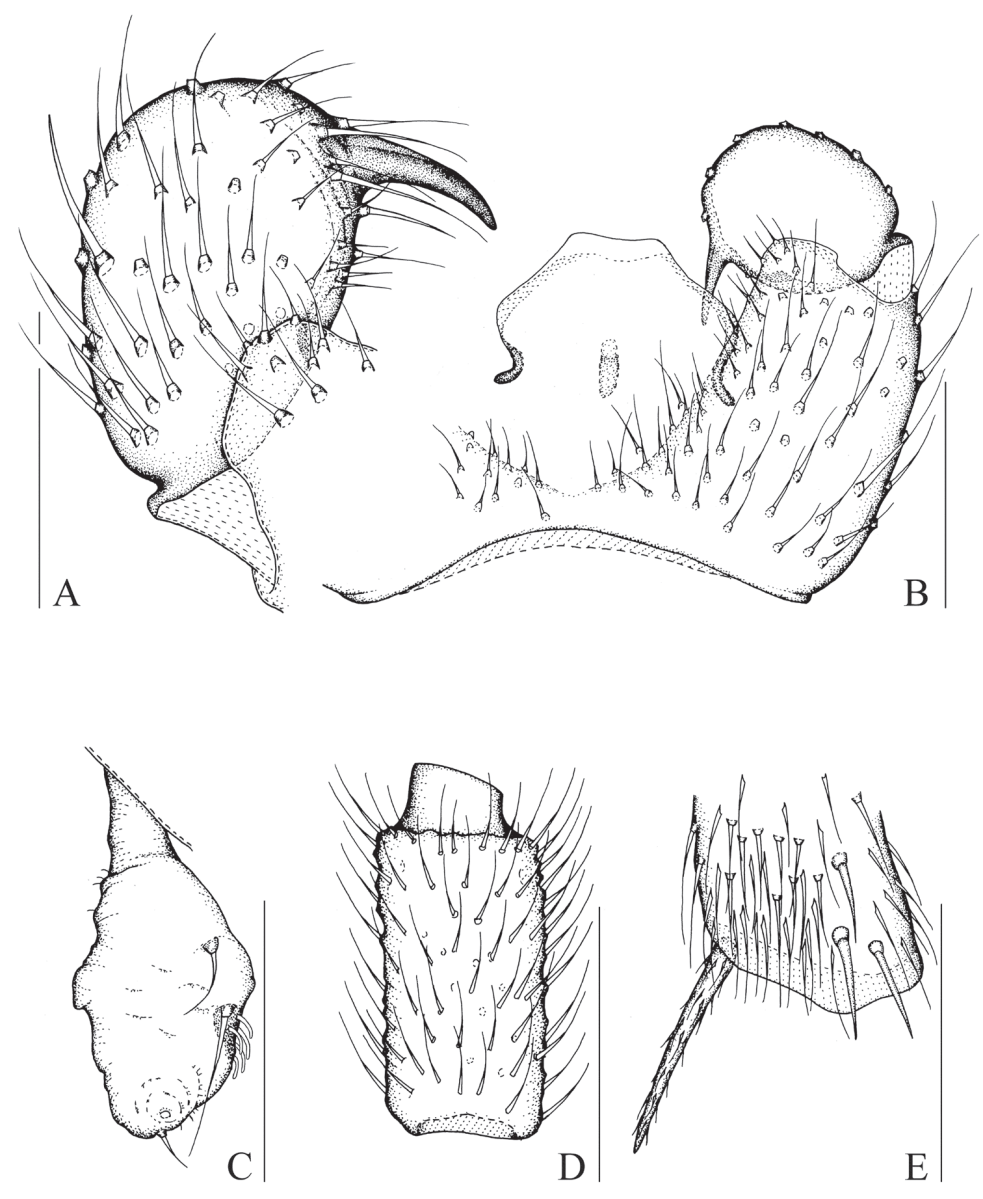
*Peyerimhoffia obesa* Shi & Huang, sp. n., male, holotype. **A** Left gonostylus, ventral view **B** Part of hypopygium, ventral view **C** Palp, lateral view **D** Fourth flagellomere, lateral view **E** Apex of foretibia, prolateral view. Scale, 0.10 mm.

Head dark brown; antenna and thorax brown; palp, abdomen, and hypopygium pale brown; legs yellowish-brown; wings fumose. **Head** (Fig. 2C, D). Eye bridge with 3 rows of facets. Prefrons with 3 setae. Clypeus non-setose. Maxillary palp 1-segmented, with 4 setae. Length/width of fourth flagellomere: 1.92. **Thorax.** Anterior pronotum with 6 setae, episternum 1 with 3 setae. **Wings** (Fig. 8B). Wing length 1.81 mm, width/length: 0.39. c/w: 0.73. R1/R: 0.70. M, Cu, stM, and r-m non-setose. **Legs** (Fig. 2E). Front tibia with a non-bordered prolateral patch of modified setae. Length of spur/width of foretibia 1.15. Length of femur/length of metatarsus: foreleg 1.29. Length of metatarsus/length of tibia: foreleg 0.51, hind leg 0.46. Length of hind tibia/length of thorax 1.32. **Hypopygium** (Fig. 2A, B). Sternite 10 with one seta on each half.

##### Distribution.

China, Shanxi (Fig. 9).

##### Remarks.

Based on the form of the gonostylus, the new species is similar to *Peyerimhoffia alpina* (Mohrig 1978). However, the new species differs in having palp is 1-segmented, the tegmen is strongly curved and greatly sclerotized, and the intercoxal area is simple in *Peyerimhoffia obesa*. In contrast, in *Peyerimhoffia alpina*, the palp is 3-segmented, the tegmen is slightly curved and weakly sclerotized, and the intercoxal area bears a lobe-like projection.

##### Etymology.

This species is named after its globally inflated gonostylus (Latin adjective *obesus* = obese).

#### Peyerimhoffia vagabunda

(Winnertz, 1867)

http://species-id.net/wiki/Peyerimhoffia_vagabunda

Sciara vagabunda Winnertz, 1867: 230.
Peyerimhoffia brachyptera Kieffer, 1903: 198.
Peyerimhoffia alata Frey, 1948: 72, 88.
Plastosciara (Peyerimhoffia) brachyptera (Kieffer, 1903): Tuomikoski 1960: 40, 41.
Cratyna (Peyerimhoffia) vagabunda (Winnertz, 1867): Menzel and Mohrig 2000: 285, 286.

##### New materials.

CHINA. HEILONGJIANG. 1 male, Haerbin, Shangzhi, Maoershan Nature Park, sweep-net, 26.VII.2008, Su-Jiong Zhang [SM00193]. SHAANXI. 1 male, Huxian, Laoyu, Baliping Zhuque Nature Park, sweep-net, 13.VII.2012, Junhao Huang [SM01656]. SHANXI. Qinshui: 2 males, Xiachuancun, Putonggou, yellow trap, 24.VII.2012, Kai Shi [SM017760–1777]; 1 male, Xiachuancun, Zhuweigou, sweep-net, 23.VII.2012, Kai Shi [SM01738]; 1 male, Xiachuancun, Zhuweigou, sweep-net, 25.VII.2012, Kai Shi [SM01770]; 1 male, Dahecun, Nanshenyu, sweep-net, 28.VII.2012, Kai Shi [SM01780]. ZHEJIANG. 1 male, Linan, Mt. Qingliangfeng, Qianqingtang, malaise trap, 15.V.2012 [SM01719].

##### Diagnosis.

The species is characterized by the 1-segmented palp, the gonostylus evenly narrowed toward the apex, the apical tooth as long as the width of the gonostylus, and the tegmen very strongly curved and sclerotized.

##### Distribution.

China (Heilongjiang, Shaanxi, Shanxi, Zhejiang – new record) (Fig. 6); Finland, Sweden, Italy, Russia (Primorsky Kray).

##### Remarks.

This species is new to China. The Chinese specimens examined show no obvious differences.

#### Peyerimhoffia sparsula

Shi & Huang sp. n.

http://zoobank.org/41E8EFCF-E77D-4802-828B-127614361356

http://species-id.net/wiki/Peyerimhoffia_sparsula

Figs 3 , 8C , 9

##### Specimens examined.

*Holotype*, male. CHINA. Shaanxi Province, Huxian, Laoyu, Baliping Zhuque Nature Park, sweep-net, 12.VII.2012, Kai Shi [SM01712].

##### Description (male).

**Figure 3.**
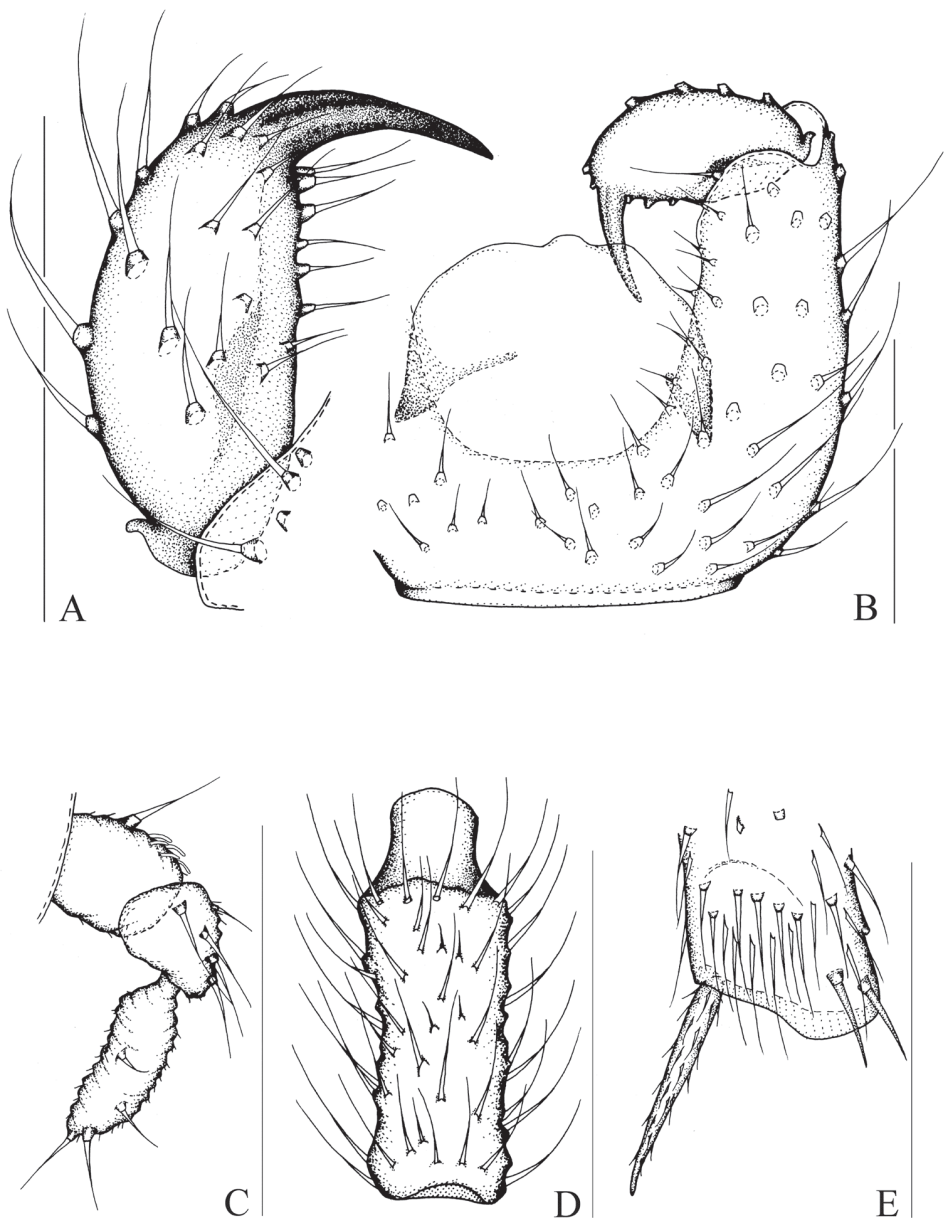
*Peyerimhoffia sparsula* Shi & Huang, sp. n., male, holotype. **A** Left gonostylus, ventral view **B** Part of hypopygium, ventral view **C** Palp, lateral view **D** Fourth flagellomere, lateral view **E** Apex of foretibia, prolateral view. Scale, 0.10 mm.

Head dark brown; antenna, thorax, abdomen, and hypopygium brown; palp pale brown; legs yellowish-brown; wings fumose. **Head** (Figs 3C, D). Eye bridge with 3 rows of facets. Prefrons with 5 setae. Clypeus with 1 seta. Maxillary palp 3-segmented, segment 1 with one seta. Length/width of fourth flagellomere: 2.30. **Thorax.** Anterior pronotum with 2 setae, episternum 1 with 4 setae. **Wings** (Fig. 8C). Wing length 1.46 mm, width/length: 0.39. c/w: 0.54. R1/R: 0.61. M, Cu, stM, and r-m non-setose. **Legs** (Fig. 3E). Front tibia with proximally bordered prolateral patch of modified setae. Length of spur/width of foretibia 1.28. Length of femur/length of metatarsus: foreleg 1.48. Length of metatarsus/length of tibia: foreleg 0.52, hind leg 0.50. Length of hind tibia/length of thorax 1.29. **Hypopygium** (Fig. 3A, B). Sternite 10 with one seta on each half.

##### Distribution.

China, Shaanxi (Fig. 9).

##### Remarks.

Based on the form of the gonostylus, the new species is similar to *Peyerimhoffia ultima* Vilkamaa & Hippa, 1998. However, the new species can be distinguished by the front tibia with a proximally bordered prolateral patch of modified setae, the gonostylus with few short subapical setae, and the tegmen sub-truncate apically. In contrast, *Peyerimhoffia ultima* has an indistinct row of setae on the front tibia, a gonostylus with numerous long subapical setae, and a tegmen moderately curved apically.

##### Etymology.

This species is named after its sparse setosity on the gonocoxite (Latin adjective *sparsulus* = sparse).

#### Peyerimhoffia longiprojecta

Shi & Huang sp. n.

http://zoobank.org/82672F3F-A9D4-4D7E-BCE8-9802B01E9CBF

http://species-id.net/wiki/Peyerimhoffia_longiprojecta

Figs 4 , 8D , 9

##### Specimens examined.

*Holotype*, male. CHINA. Shanxi Province, Qinshui, Xiachuancun, Putonggou, sweep-net, 24.VII.2012, Kai Shi [SM01737]. *Paratype*, SHANXI. 1 male, the same data as holotype [SM01736].

##### Description (male).

**Figure 4.**
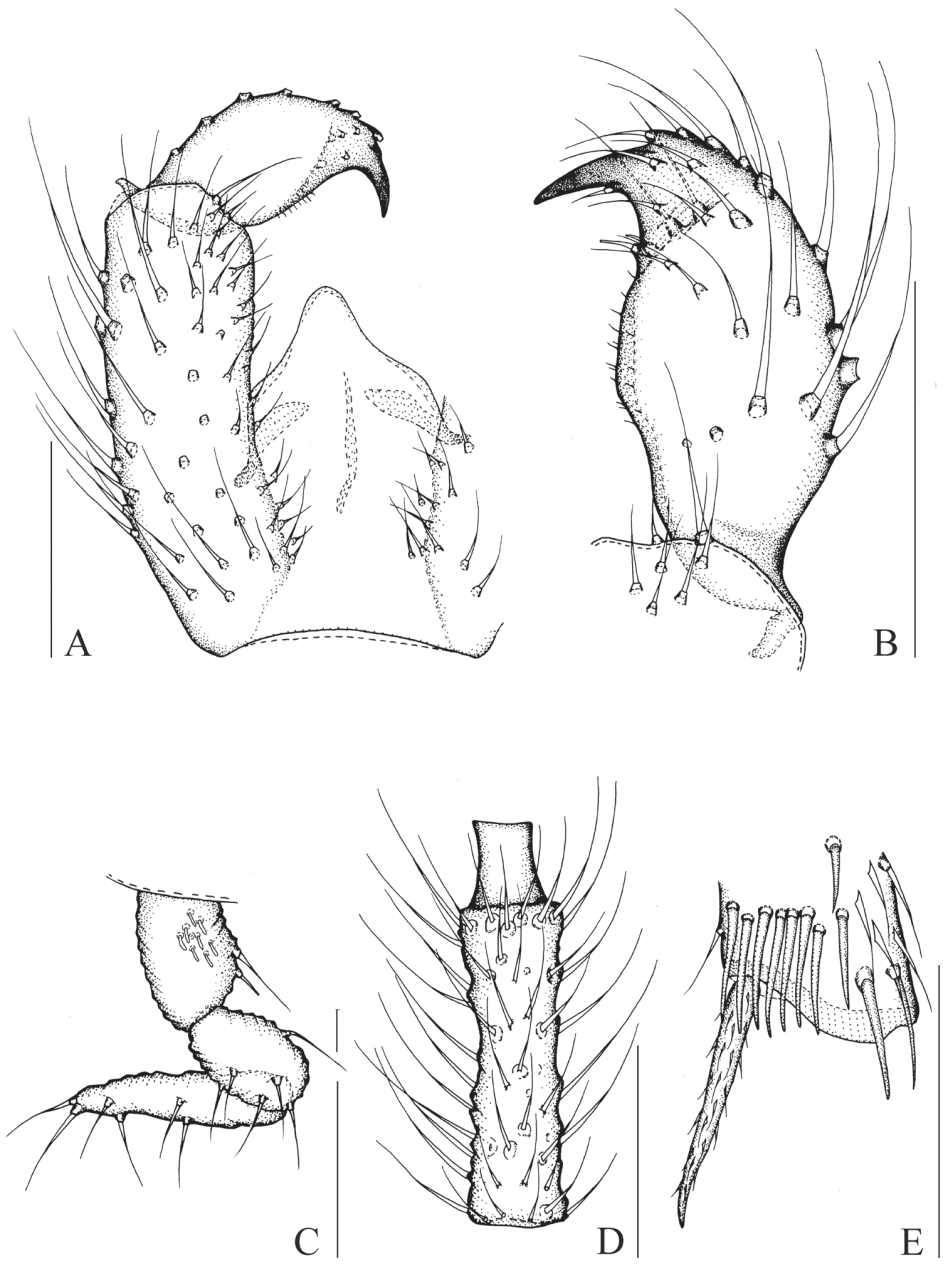
*Peyerimhoffia longiprojecta* Shi & Huang, sp. n., male, holotype. **A** Part of hypopygium, ventral view **B** Right gonostylus, ventral view **C** Palp, lateral view **D** Fourth flagellomere, lateral view **E** Apex of foretibia, prolateral view. Scale, 0.10 mm.

Head dark brown; antenna, thorax, and abdomen brown; palp and hypopygium pale brown; legs yellowish-brown; wings fumose. **Head** (Fig. 4C, D). Eye bridge with 3 rows of facets. Prefrons with 7–8 setae. Clypeus non-setose. Maxillary palp 3-segmented, segment 1 with 2 setae. Length/width of fourth flagellomere: 3.86–4.07. **Thorax.** Anterior pronotum with 2 setae, episternum 1 with 3 setae. **Wings** (Fig. 8D). Wing length 1.80 mm, width/length: 0.39. c/w: 0.81. R1/R: 0.43. M, Cu, stM, and r-m non-setose. **Legs** (Fig. 4E). Front tibia with an indistinct row of seven spinose setae. Length of spur/width of foretibia 1.27–1.39. Length of femur/length of metatarsus: foreleg 1.34–1.43. Length of metatarsus/length of tibia: foreleg 0.46–0.50, hind leg 0.46–0.47. Length of hind tibia/length of thorax 1.40–1.35. **Hypopygium** (Fig. 4A, B). Sternite 10 with one seta on each half.

##### Distribution.

China, Shanxi (Fig. 9).

##### Remarks.

This species is unique within the genus in having its tegmen greatly projected in the middle of the apical margin. Based on the form of the gonostylus, it is similar to *Peyerimhoffia menzeli* Vilkamaa & Hippa, 2005, but differs in having a very slender fourth flagellomere that is about four times longer than its width and a tegmen that is strongly projected apically. In contrast, in *Peyerimhoffia menzeli*, the fourth flagellomere is about twice longer than its width and the tegmen is truncate apically.

##### Etymology.

This species is named after the great middle projection of the apical tegmen (Latin adjective *longiprojecta* = long projection).

#### Peyerimhoffia brachypoda sp. n.

http://zoobank.org/E39D802B-D633-44EE-A527-83B4E39B6828

http://species-id.net/wiki/Peyerimhoffia_brachypoda

Figs 5 , 8E , 9

##### Specimens examined.

*Holotype*, male. CHINA. Zhejiang province, Anji, Mt. Longwangshan, sweep-net, 31.III.2012, Kai Shi [SM01588]. *Paratype*, SHANXI. 1 male, Qinshui, Dongchuancun, Dongxia, sweep-net, 25.VII.2012, Kai Shi [SM01791].

##### Description (male).

**Figure 5.**
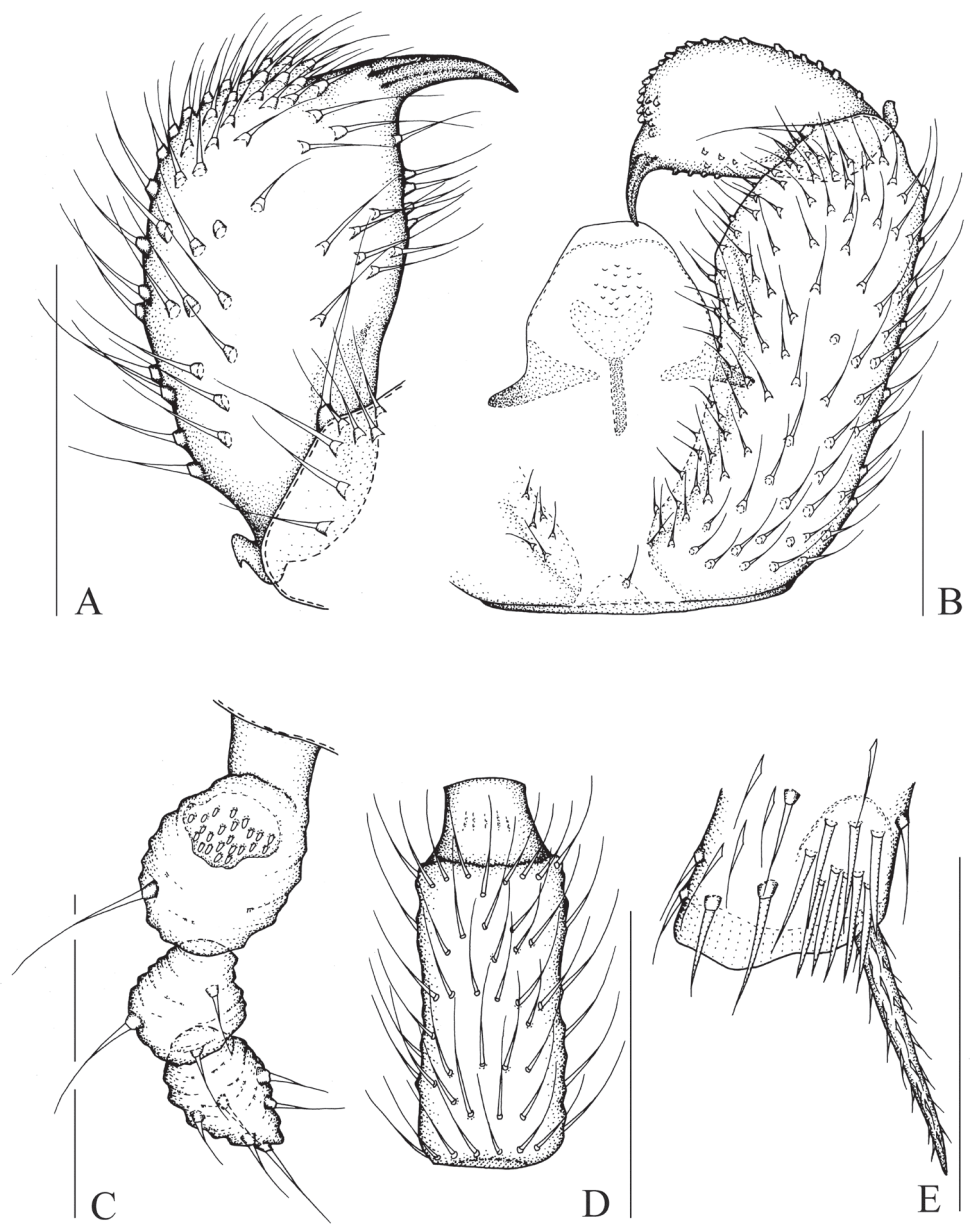
*Peyerimhoffia brachypoda* Shi & Huang, sp. n., male, holotype. **A** Left gonostylus, ventral view **B** Part of hypopygium, ventral view **C** Palp, lateral view **D** Fourth flagellomere, lateral view **E** Apex of foretibia, prolateral view. Scale, 0.10 mm.

Head dark brown; antenna, thorax, abdomen and hypopygium brown; palp pale brown; legs yellowish-brown; wings fumose. **Head** (Fig. 5C, D). Eye bridge with 2 rows of facets. Prefrons with 4 setae. Clypeus with 2 setae. Maxillary palp 3-segmented, segment 1 with 1–2 setae. Length/width of 4th flagellomere: 2.36–2.74. **Thorax.** Anterior pronotum with 2 setae, episternum 1 with 4–5 setae. **Wings** (Fig. 8E). Wing length 1.34–1.46 mm, width/length: 0.41–0.44. c/w: 0.52–0.55. R1/R: 0.51–0.53. M, Cu, stM and r-m nonsetose. **Legs** (Fig. 5E). Front tibia with bordered prolateral patch of few strong modified setae. Length of spur/width of foretibia 1.13–1.19. Length of femur/length of metatarsus: foreleg 1.37–1.75. Length of metatarsus/length of tibia: foreleg 0.51–0.54, hind leg 0.45–0.49. Length of hind tibia/length of thorax 1.47–1.50. **Hypopygium** (Fig. 5A, B). Sternite 10 with one seta on each half.

##### Distribution.

China (Shanxi, Zhejiang, Fig. 9).

##### Remarks.

The new species is similar to *Peyerimhoffia infera* Vilkamaa & Hippa, 2005 in the shape of the gonostylus. However, the new species can be distinguished by the three-segmented maxillary palp, the gonostylus having much inflated apex and short subapical setae (two-segmented maxillary palp, the gonostylus slightly inflated at apex and bearing long subapical setae in *Peyerimhoffia infera*).

##### Etymology.

This species is named in reference to its short apical lobe, from theGreek adjective *brachypodus*, meaning short base.

#### Peyerimhoffia yunnana sp. n.

http://zoobank.org/7FEE26A1-7F74-462F-8C00-6E045E6DC32E

http://species-id.net/wiki/Peyerimhoffia_yunnana

Figs 6 , 8F , 9

##### Specimens examined.

*Holotype*, male. CHINA. Yunnan province, Honghe, Lvcun, Mt. Huanglianshan, Yakou, 1950 m, sweep-net, 8.V.2011, Yan Li [SM01589].

##### Description (male).

**Figure 6.**
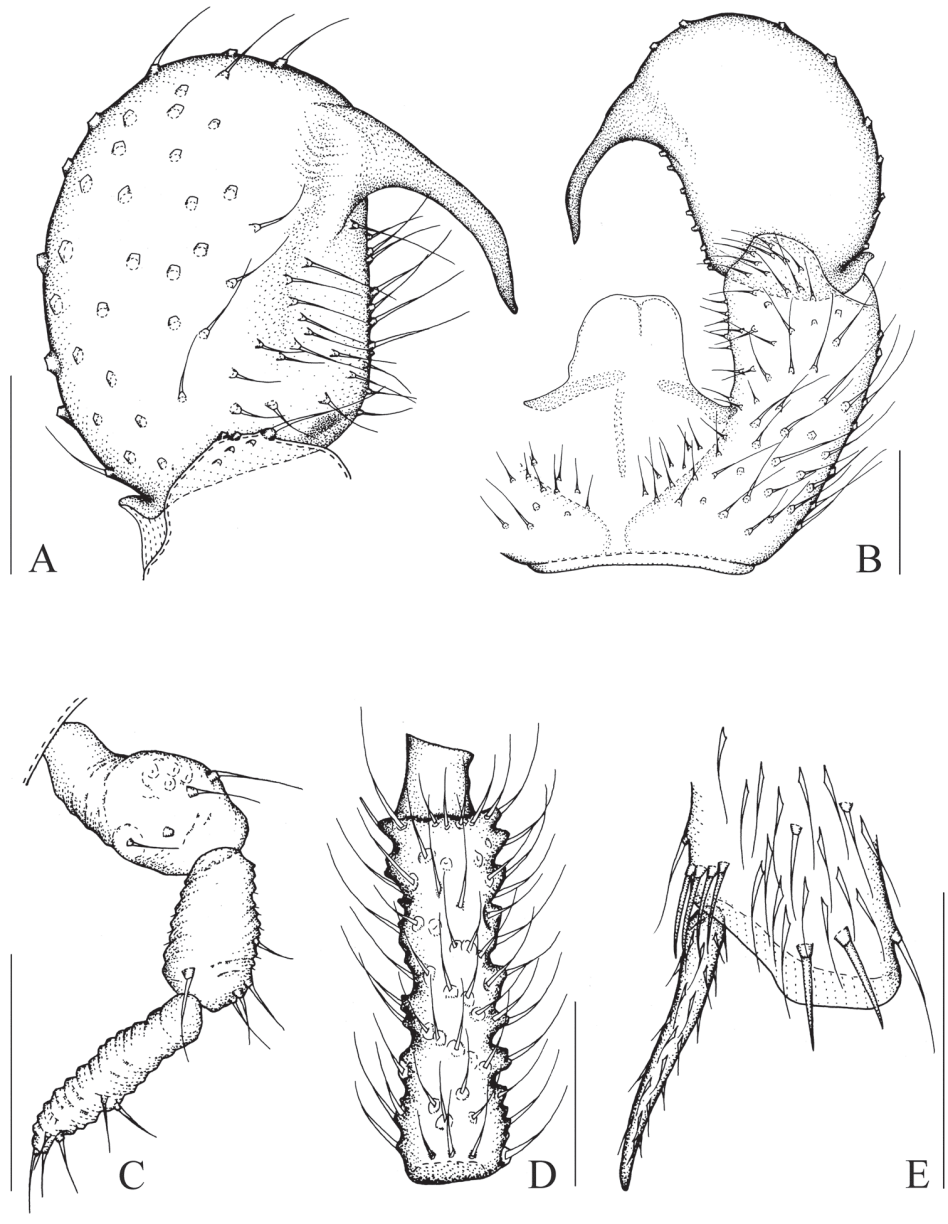
*Peyerimhoffia yunnana* Shi & Huang, sp. n., male, holotype. **A** Left gonostylus, ventral view **B** Part of hypopygium, ventral view **C** Palp, lateral view **D** Fourth flagellomere, lateral view **E** Apex of foretibia, prolateral view. Scale, 0.10 mm.

Head dark brown; antenna, thorax, abdomen and hypopygium brown; palp pale brown; legs yellowish-brown; wings fumose. **Head** (Fig. 6C, D). Eye bridge with 3 rows of facets. Prefrons with 11 setae. Clypeus with 1 seta. Maxillary palp 3-segmented, segment 1 with 4 setae. Length/width of 4th flagellomere: 4.37. **Thorax.** Anterior pronotum with 6 setae, episternum 1 with 4 setae. **Wings** (Fig. 8F). Wing length 2.06 mm, width/length: 0.44. c/w: 0.59. R1/R: 0.67. M, Cu, stM and r-m nonsetose. **Legs** (Fig. 6E). Front tibia with non-bordered prolateral patch of 4 modified setae in low. Length of spur/width of foretibia 1.38. Length of femur/length of metatarsus: foreleg 1.34. Length of metatarsus/length of tibia: foreleg 0.54, hind leg 0.49. Length of hind tibia/length of thorax 1.68. **Hypopygium** (Fig. 6A, B). Sternite 10 with one seta on each half.

##### Distribution.

China (Yunnan, Fig. 9).

##### Remarks.

This species is unique in having the light-colored gonostylar apical tooth, which is not scleritised.

##### Etymology.

This species is named after its type locality, Yunnan province, China.

#### Peyerimhoffia shennongjiana sp. n.

http://zoobank.org/8F2367D5-2651-4AC4-A181-983F09875DCB

http://species-id.net/wiki/Peyerimhoffia_shennongjiana

Figs 7 , 8G , 9

##### Specimens examined.

*Holotype*, male. CHINA. Hubei province, Shennongjia, Dalongtan, sweep-net, 20.V.2012, Kai Shi [SM01662].

##### Description (male).

**Figure 7.**
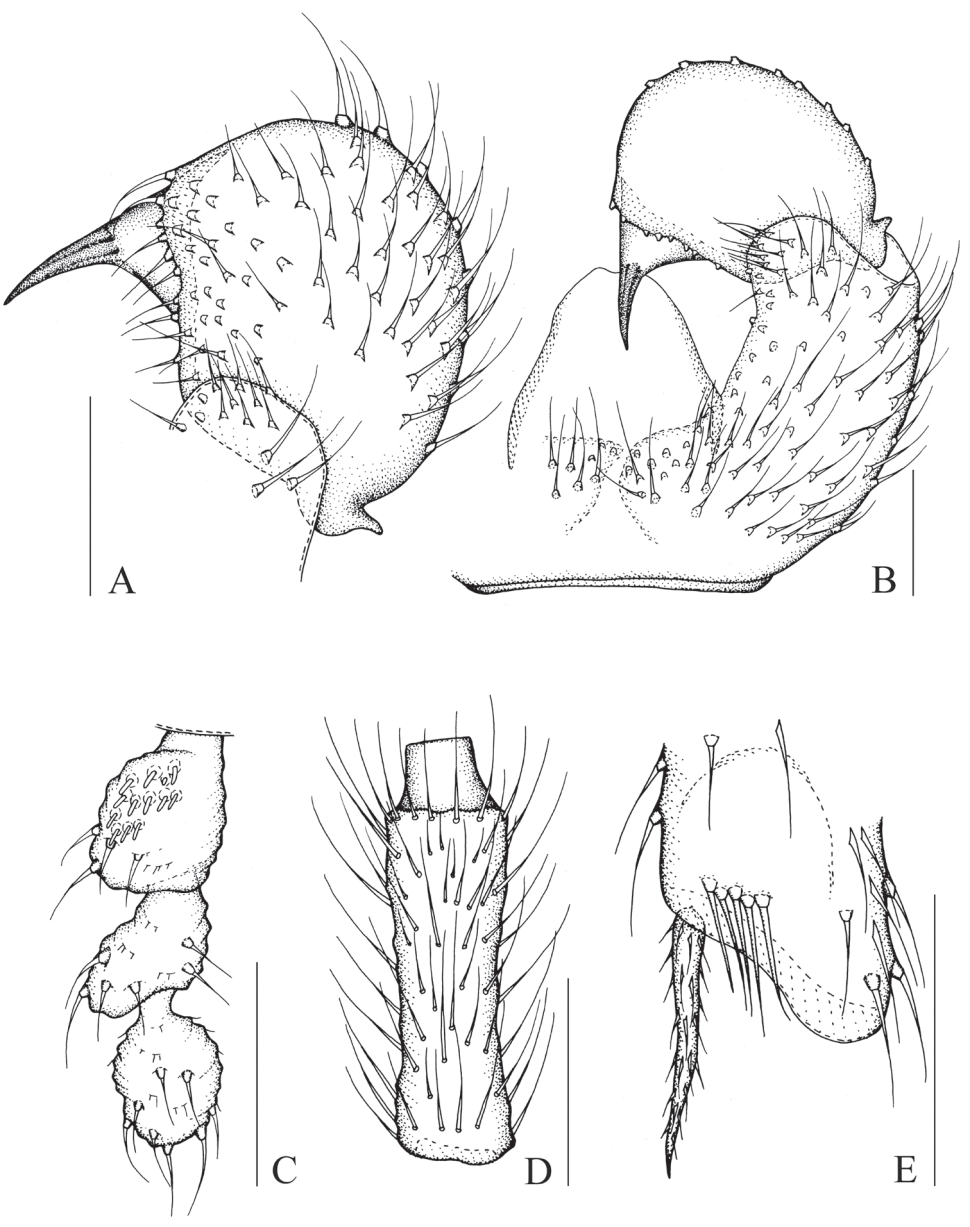
*Peyerimhoffia shennongjiana* Shi & Huang, sp. n., male, holotype. **A** Right gonostylus, ventral view **B** Part of hypopygium, ventral view **C** Palp, lateral view **D** Fourth flagellomere, lateral view **E** Apex of foretibia, prolateral view. Scale, 0.10 mm.

**Figure 8.**
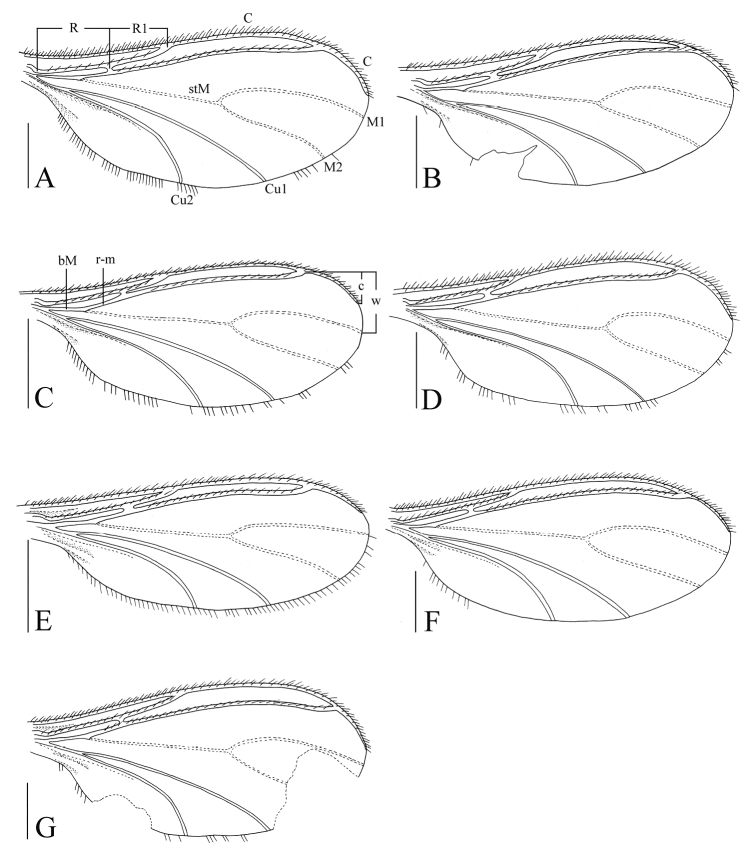
Wings, dorsal view. **A** *Peyerimhoffia hamata* sp. n. (holotype) **B** *Peyerimhoffia obesa* sp. n. (holotype) **C** *Peyerimhoffia sparsula* sp. n. (holotype) **D** *Peyerimhoffia longiprojecta* sp. n. (holotype) **E** *Peyerimhoffia brachypoda* sp. n. (holotype) **F** *Peyerimhoffia yunnana* sp. n. (holotype) **G** *Peyerimhoffia shennongjiana* sp. n. (holotype). Scale, 0.50 mm.

Head dark brown; palp, antenna, thorax, abdomen and hypopygium brown; legs yellowish-brown; wings fumose. **Head** (Fig. 7C, D). Eye bridge with 3 rows of facets. Prefrons with 26 setae. Clypeus with 1 seta. Maxillary palp 3-segmented, segment 1 with 4 setae. Length/width of 4th flagellomere: 2.83. **Thorax.** Anterior pronotum with 2 setae, episternum 1 with 4 setae. **Wings** (Fig. 8G). Wing length 2.25 mm, width/length: 0.46. c/w: 0.79. R1/R: 0.88. M, Cu and stM nonsetose. r-m with 4 setae. **Legs** (Fig. 7E). Front tibia with bordered prolateral patch of 5 modified setae. Length of spur/width of foretibia 1.06. Length of femur/length of metatarsus: foreleg 1.69. Length of metatarsus/length of tibia: foreleg 0.48, hind leg 0.50. Length of hind tibia/length of thorax 1.45. **Hypopygium** (Fig. 7A, B). Sternite 10 with one seta on each half.

##### Distribution.

**Figure 9.**
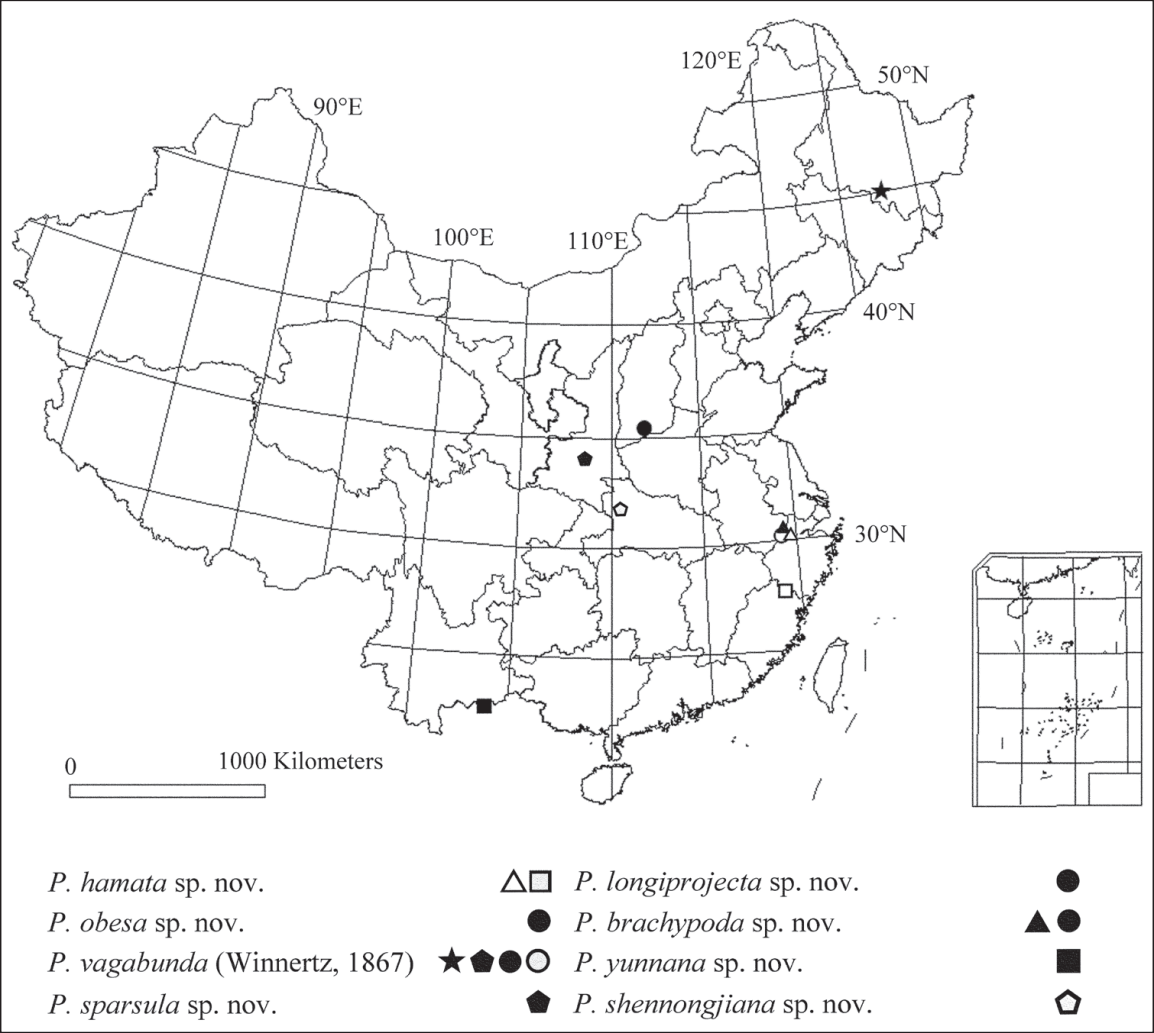
Geographical distribution of *Peyerimhoffia* from China.

China (Hubei, Fig. 9).

##### Remarks.

This species can be readily recognized by having two unique characteristics: the gonostylar apex bare, and a dorsally located lobe on its inflated gonostylus.

##### Etymology.

This species is named after its type locality, Shennongjia at Hubei province, China.

## Supplementary Material

XML Treatment for Peyerimhoffia hamata

XML Treatment for Peyerimhoffia obesa

XML Treatment for Peyerimhoffia vagabunda

XML Treatment for Peyerimhoffia sparsula

XML Treatment for Peyerimhoffia longiprojecta

XML Treatment for Peyerimhoffia brachypoda

XML Treatment for Peyerimhoffia yunnana

XML Treatment for Peyerimhoffia shennongjiana
